# The regulatory effect and molecular mechanism of lncRNA Gm10451 on islet cell dysfunction in children with diabetes

**DOI:** 10.3389/fgene.2022.927471

**Published:** 2022-08-08

**Authors:** Jiao Wang, Li-hai Zhang, Yu-ming Kang, Xian-he Wang, Chun-yu Jiang

**Affiliations:** ^1^ Department of Physiology and Pathophysiology, Xi’an Jiaotong University School of Basic Medical Sciences, Xi’an, China; ^2^ The First Affiliated Hospital of Jiamusi University, Jiamusi, Heilongjiang, China

**Keywords:** Gm10451 causes islet cell dysfunction lncRNA, Gm10451, islet, diabetes, insulin

## Abstract

The dysfunction of islet *β-*cells is one of the causes of diabetes, and lncRNA Gm10451 is also a participant in the occurrence and the development of various diseases. This study was carried out to reveal the correlation within *β-*cells and Gm10451. Our study was started with the cellular cultivation of MIN6 cells *in vitro*, where this islet *β-*cell line was randomly divided into the groups of control, hyperglycemia, Gm10451 siRNA tansfection, and Gm10451 tansfection. Of all these treatments, cells in the groups of Gm10451 siRNA tansfection and Gm10451 tansfection were given with lentiviral transfection under hyperglycemia condition. Further explorations were established using PCR assay and MTT method to evaluate Gm10451 expression and estimate cellular proliferation. It ended up with the enzyme-linked immunosorbent assay (ELISA) to assess Caspase 3 activity, superoxide dismutase (SOD) activity, and reactive oxygen species (ROS) content and the secretion of IL-10 and IL-1. It was found that Gm10451 expression in MIN6 cells under hyperglycemia cultivation was notably higher than the control group; likewise, a transfection with the lentivirus of Gm10451 also resulted in the upregulation of Gm10451 expression, succeeded with inhibiting cellular proliferation, enhancing Caspase 3 activity, and decreasing SOD activity. In the lentivirus transfection groups, transfection of Gm10451 elevated the ROS content and promoted IL-1 expression, and it also decreased both IL-10 expression and insulin secretion, leading to a consequence of statistically significant difference in contrast to the high-glucose group; on the contrary, transfection of Gm10451 siRNA in a high-glucose environment downregulated the expression of Gm10451 and inversed those change before, whose results were statistically significant when compared with the high-glucose group. Hyperglycemia promotes the expression of Gm10451. Targeting inhibition toward Gm10451 alleviates cellular apoptosis and the oxidative stress of islet cells, promoting proliferation and insulin secretion of islet cells.

## Highlights

Hyperglycemia promoted Gm10451 expression in MIN6 cells
Gm10451 inhibited MIN6 cell proliferation.
Gm10451 promoted MIN6 cell apoptosis
Gm10451 promoted the secretion of inflammatory factors
Gm10451 inhibited insulin secretion of the MIN6 cells

## Introduction

Diabetes is not only a common endocrine system metabolic disease but also one of the world’s health problems, with high morbidity and complications. Diabetes is caused by hyperglycemia caused by deficiency of insulin secretion or dysfunction of islet secretion (de Goede et al., 2021; Radhakrishnan and Kowluru, 2021). The long-term hyperglycemia caused by diabetes will lead to a variety of complications and cause multiple organ damages. The pathogenesis of diabetes is complex, among which, disorder of glucose and lipid metabolism, inflammation, oxidative stress, and apoptosis can be considered as the main factors for its occurrence and development. Second, diabetes is also influenced by many factors, including genetics, autoimmune defects, and viral infections. Normally, on the cellular level, dysfunction of the islet *β-*cells leads to a reduction in insulin production, leading to diabetes (Lodde et al., 2020; Stanzione et al., 2020; Yang et al., 2020).

Recent research evidence suggested that diabetes is also closely related to inflammation of the islet caused by immune disorders (Li J et al., 2021; Hao et al., 2021). In this context, inflammatory factors are central to diabetes caused by immune disorders; in other words, the islet *β*-cell apoptosis and the insulin deficiency induced by these inflammatory factors are key to the course of diabetes (Thomas et al., 2019; Casado-Diaz et al., 2020; Zhang et al., 2021).

lncRNAs are the transcripts with more than 200 nucleotides in length. Although they are non-protein-coding RNAs, they biologically function in a way of transcription or post-transcription and are indispensable regulatory factors in the occurrence and development of diseases and the biological process (Thomas et al., 2019; Casado-Diaz et al., 2020; Zhang et al., 2021). Since there is no relevant study on Gm10451 and diabetes, this study focused on Gm10451 and the islet β-cells cultured in the hyperglycemia environment and evaluated the effect of lncRNA on islet β cells. In order to clarify the regulatory effect of Gm10451 on the proliferation and secretion of pancreatic *β* cells, this study over-expressed and interfered with Gm10451 in MIN6 islet cells cultured *in vitro*. EDU staining was used to detect cell proliferation. TUNEL staining was used to detect cell apoptosis, and ELISA was used to detect secretory function of cells. This study provides a new research basis for the application of Gm10451 in diabetes.

## Material and methods

### Cell cultivation and transfection

MIN6 islet cells were purchased from ATCC, and they were cultured with the RPMI-1640 medium, containing 10% FBS, formulated with 10 U/mL penicillin and 10 μg/ml streptomycin (Hyclone, United States) in a 37 °C, 5% CO_2_ incubator. MIN6 cells in the logarithmic growth phase of the third-generation were randomly divided into three groups: control group (normal cultured cells), hyperglycemia group (30 mmol/L glucose was used to prepare hyperglycemic environment to stimulate cultured cells), and Gm10451 siRNA group and Gm10451 over-expressed group (transfection of Gm10451 siRNA and Gm10451 lentivirus were conducted under hyperglycemia, respectively). Gm10451 siRNA and Gm10451 lentivirus were added into serum-free medium, and fully mixed, and incubated at room temperature. Mixture of Lipo2000 was mixed with the corresponding diluent and placed at room temperature for 30 min, with which the MIN6 cells were cultured in a 5% CO_2_ incubator.

### qPCR assay

The total RNA was extracted from MIN6 islet cells, using the TRIzol reagent, and cDNA reverse transcription was performed according to the kit instructions (TaKaRa, Japan). The reaction conditions of qPCR were as follows: 55°C for 1 min, 92°C for 30 s, 58°C for 45 s, and 72°C for 30 s, a total of 35 cycles, and the reference gene was GAPDH. The results were analyzed semi-quantitatively by the 2^−ΔC^ method.

### MTT method detection on cellular proliferation

MIN6 cell lines at the logarithmic growth stage were digested, counted, and transferred to 96-well plates. According to the aforementioned treatment, five multiple holes were designed for each group. After adding related factors according to the group assignment, 20 μL 5 g/L MTT solution was add to each well, and then the cells were incubated in the incubator for 4 h. Then, 150 μL/well DMSO was added to each well, and the cell proliferation rate was reckoned. After the purple crystal was completely dissolved, the absorbance (A) was measured using a microplate reader at 492 nm.

### Cellular apoptosis detection using TUNEL method

After washing the cells with PBS, they were fixed with 4% paraformaldehyde for 30 min. After that, the cells were washed with PBS for the first time. PBS containing 0.3% Triton X-100 was added to the well and incubated with the cells at room temperature for 5 min. The cells were washed the second time with PBS. The cells were added with 50-μL TUNEL reaction mixture, and incubated at 37°C for 60 min away from light. The cells were washed with PBS for the third time. The samples were sealed with antifluorescence quenching sealing solution and observed under a fluorescence microscope.

### IL-1 and IL-10 levels were evaluated by ELISA

Supernatant of medium in cell culture plates of each group was collected to measure the secretion of inflammatory cytokines IL-1 and IL-10. The experiment was carried out according to the instructions of the ELISA kit. The determination should be made within 15 min after the addition of termination solution. The linear regression equation of the standard curve was calculated in terms of the concentration of the standard substance and the corresponding OD value, and the samples’ concentration was calculated on the regression equation according to the OD value of samples.

### Determination of SOD content

The alterations of SOD activity and ROS content in each group were detected according to the kit instructions, and the related parameters of oxidative stress index were obtained. The cell proteins were separated and washed in a 95°C water bath. After 40 min, the sample was taken out and rinsed with cold water. After cooling, the sample was centrifuged at 4,000 rpm for 10 min. The ethanol phase was extracted from tissue homogenate with ethanol–chloroform mixture (volume ratio 5:3) to detect the total SOD activity.

### ROS content determination

Alterations in ROS levels in each group were examined. The treated cells were immersed in 95°C water bath for 40 min, then removed, and washed with cold water. After cooling, the cells were centrifuged at 4,000 rpm for 10 min. Tissue homogenates were incubated with 2, 7 -dichlorofluorescein diacetate (DCF DA) at 37°C for 15 min, centrifuged at 10,000 rpm for 15 min, supernatant was discarded, and the precipitate was re-suspended in PBS and incubated at 37°C for 60 min. ROS levels were then measured by using a spectrophotometer.

### Analysis to insulin secretion level

MIN6 cells in the logarithmic growth phase of each group were counted, digested, and re-counted, and the cell density was optimized. The cells were inoculated with 500 μL/well in 48-well plates for 24 h. Then, the supernatant was removed, cells were washed with PBS, added with 300 μL/well HBSS buffer, and incubated at 37°C for 30 min. After that, the supernatant was centrifuged at 4°C at 800 rpm for 5 min and stored at -20°C. The protein content was measured by bicinchonininc acid (BCA) assay. Insulin concentration per unit mass = insulin content/corresponding protein content/well.

### Statistical analysis

SPSS 22.0 software (IBM, Armonk, NY, United States) was used for all data processing and statistical analysis. The measured data were described as mean ± standard deviation (SD). One-way ANOVA with Bonferroni postmortem test was used to compare multiple groups of samples. The *t*-test was used to compare the two groups. *p* < 0.05 was statistically significant.

## Results

### Hyperglycemia promoted the Gm10451 expression in MIN6 cells

**FIGURE 1:**
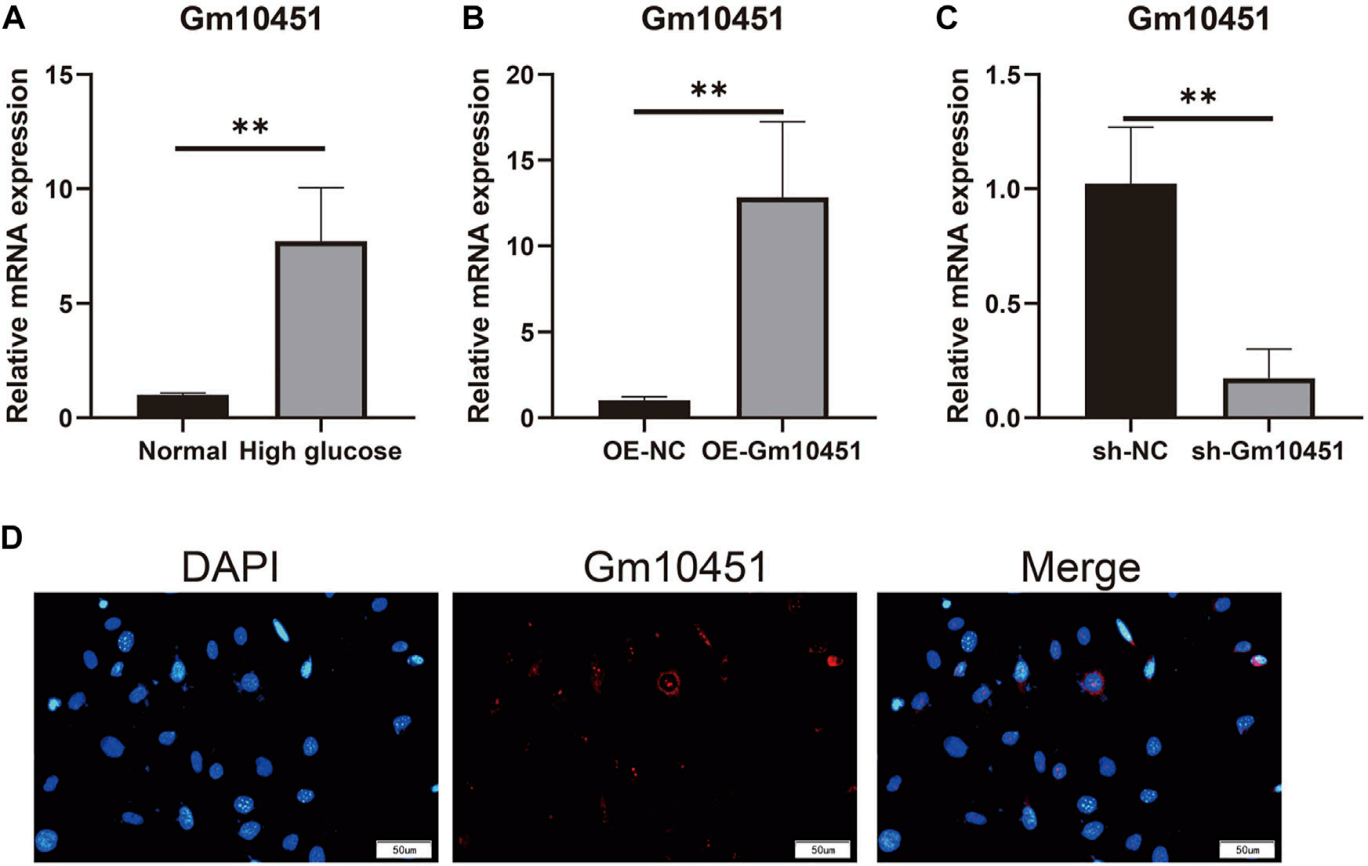
Gm10451 expression levels of MIN6 cells in the hyperglycemic environment. **(A)** Determination on Gm10451 expression in MIN6 cells under hyperglycemia by qPCR assay. **(B)** Determination on the Gm10451 expression level in Gm10451 over-expressed MIN6 cells by qPCR assay. **(C)** Determination on the Gm10451 expression level in Gm10451-interfered MIN6 cells by qPCR assay. **(D)** Gm10451s localization in MIN6 cells was pinpointed by FISH test. *N* = 3. **, *p* < 0.01.

Compared with the control group, Gm10451 expression in MIN6 cells under hyperglycemia was increased (*p* < 0.05). Gm10451 expression in MIN6 cells transfected with lentivirus Gm10451 in the hyperglycemia environment was increased (*p* < 0.05). Gm10451 siRNA was transfected into MIN6 cells in the hyperglycemia environment, whose Gm10451 expression was decreased when compared with that in the hyperglycemia group (*p* < 0.05; Figure 1).

### Gm10451 hindered MIN6 cell proliferation and promoted MIN6 cell apoptosis

**FIGURE 2:**
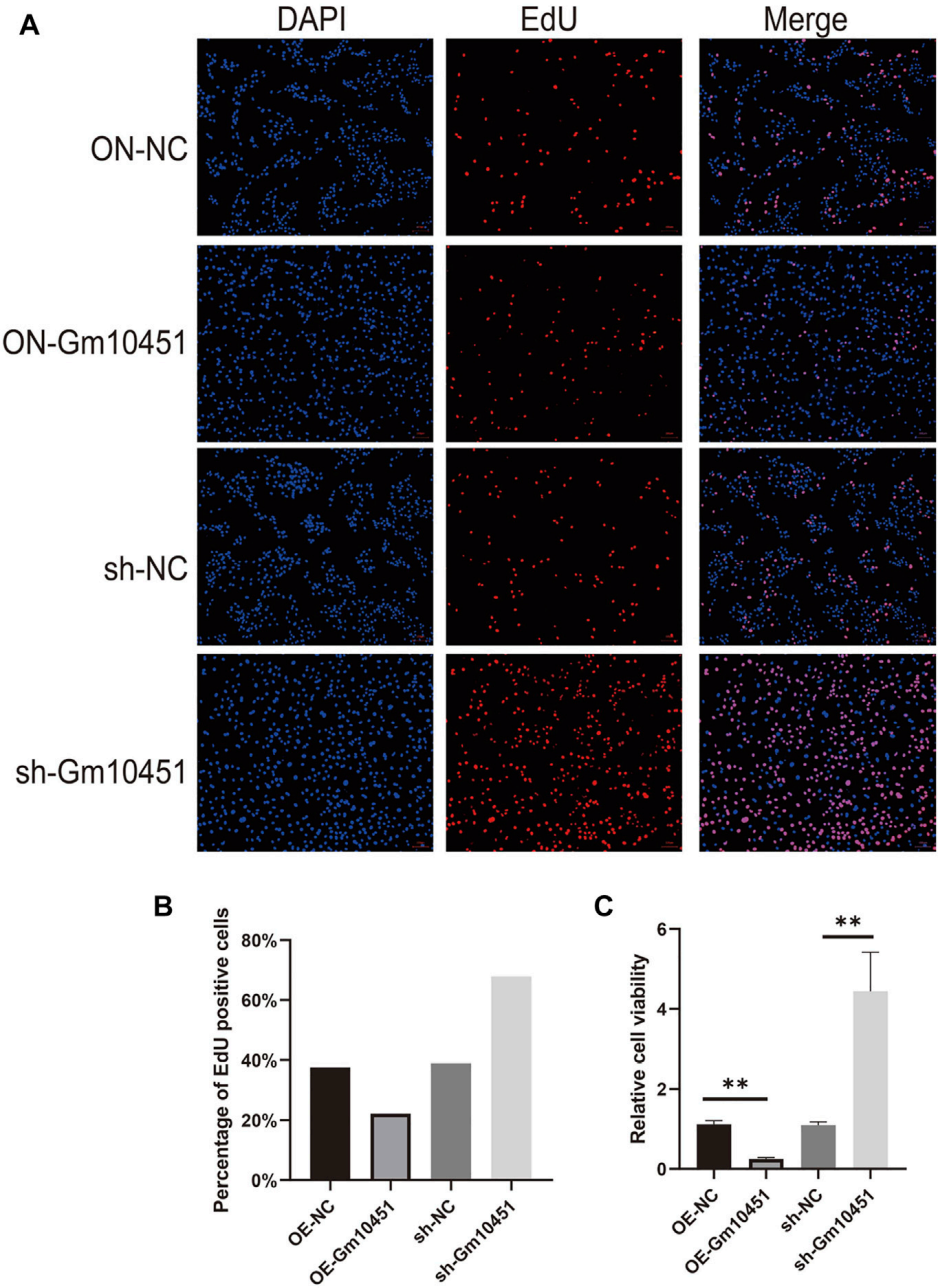
High levels of Gm10451 hindered MIN6 cell proliferation. **(A,B)** Assessment on MIN6 cell proliferation *via* Edu. **(C)** Assessment on MIN6 cell proliferation *via* MTT. *N* = 3. **, *p* < 0.01.

Edu staining was performed to detect cell proliferation. In a hyperglycemic environment, the Gm10451 lentivirus transfection into MIN6 cells further hindered MIN6 cell proliferation (the percentage of Edu positive cells was decreased), and the difference was statistically significant when compared with the hyperglycemic group (ON-NC group) (*p* < 0.05). Conversely, Gm10451 siRNA transfection into MIN6 cells promoted MIN6 cell proliferation under hyperglycemia (the percentage of Edu positive cells was increased) (*p* < 0.05; Figure 2).

**FIGURE 3:**
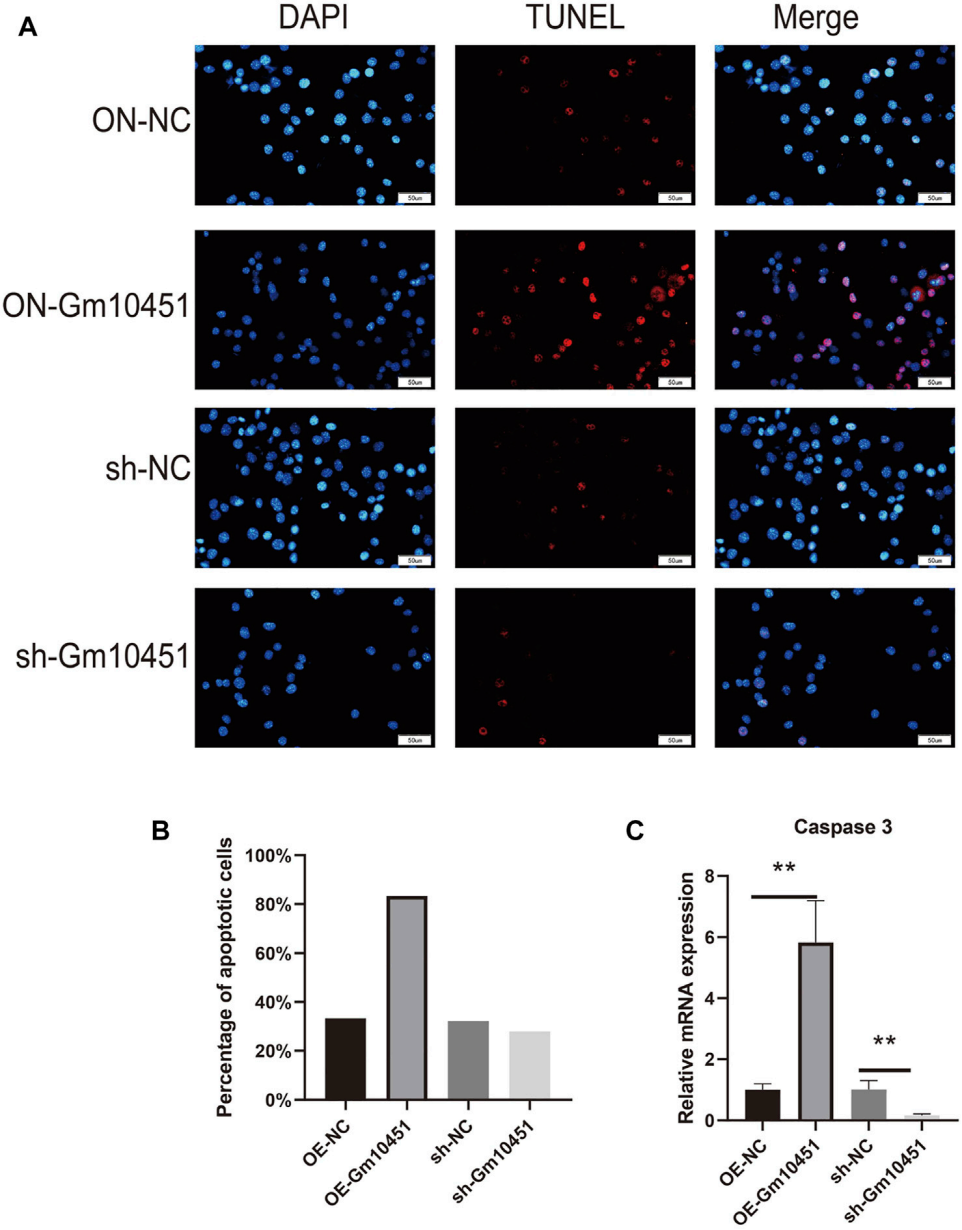
Highly expressing Gm10451 promoted MIN6 cell apoptosis. **(A,B)** Determination on apoptosis of MIN6 cells using TUNEL assay. **(C)** Determination on apoptosis-related proteins’ expression through qPCR. *N* = 3. **, *p* < 0.01.

The Caspase 3 activity and the percentage of TUNEL positive cells were increased under hyperglycemia condition. TUNEL positive cells and Caspase 3 activity of MIN6 cells were increased after transfection with Gm10451 lentivirus in hyperglycemia, when compared with the control group (ON-NC group), and the difference was statistically significant (*p* < 0.05). Transfection of Gm10451 siRNA into MIN6 cells resulted in an inhibition of Caspase 3 activity and decrease of the percentage of TUNEL positive cells (*p* < 0.05; Figure 3).

### High level of Gm10451 promoted the secretion of inflammatory factors

**FIGURE 4:**
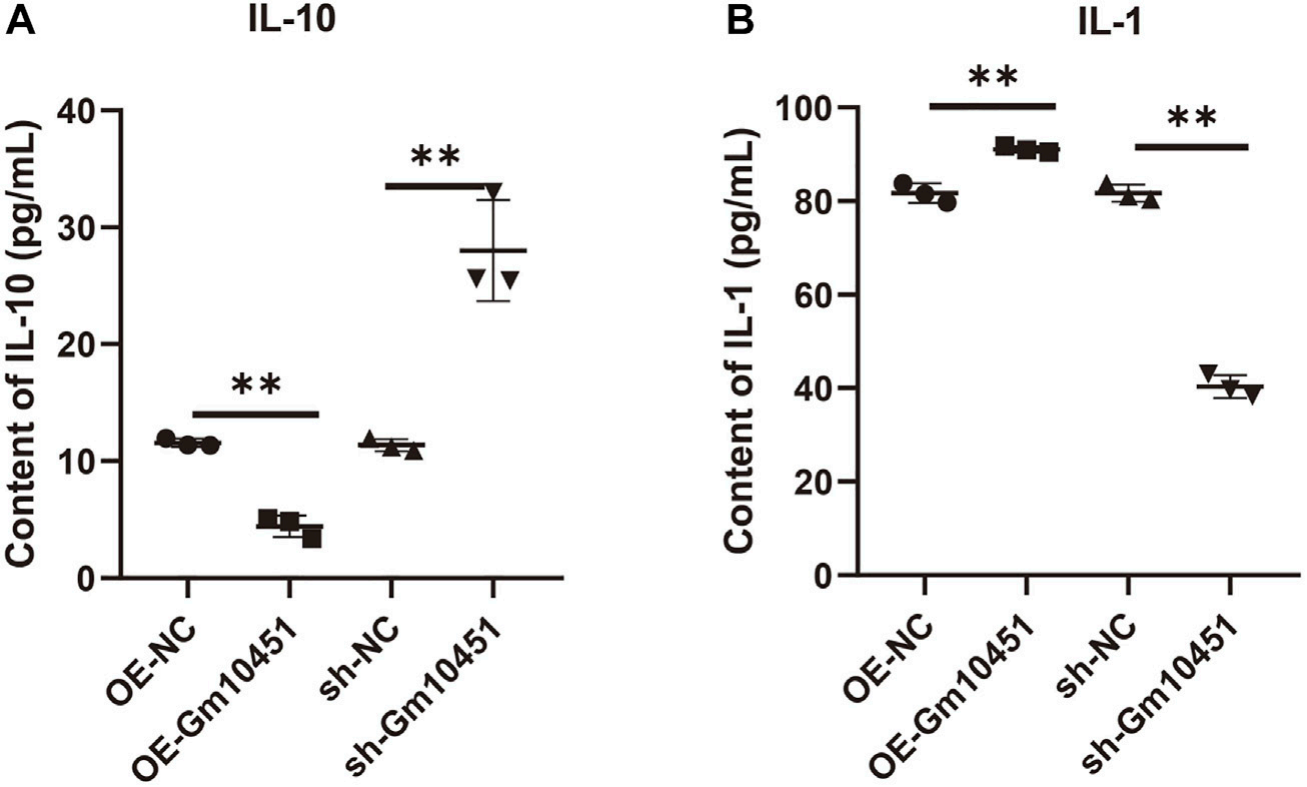
High levels of Gm10451 increased the secretion of inflammatory factors. The expression levels of IL-10 and IL-1 were determined by ELISA. **(A)** Determination on IL-10 expression using the ELISA method. **(B)** Determination on IL-1 expression using the ELISA method. *N* = 3. **, *p* < 0.01.

The effects of Gm10451 on inflammation and anti-inflammatory factor secretion of MIN6 cells cultured in hyperglycemia were evaluated by ELISA. Gm10451 lentivirus transfection into MIN6 cells reduced the secretion of IL-10 and increased the secretion of IL-1, with statistically significant difference when compared with the hyperglycemia group (ON-NC group) (*p* < 0.05). Transfection of Gm10451 siRNA into MIN6 cells increased IL-10 secretion and decreased IL-1 secretion, with statistically significant differences in contrast to the hyperglycemia group (sh-NC group) (*p* < 0.05; Figure 4).

### High levels of Gm10451 hindered insulin secretion of the MIN6 cells in hyperglycemia

**FIGURE 5:**
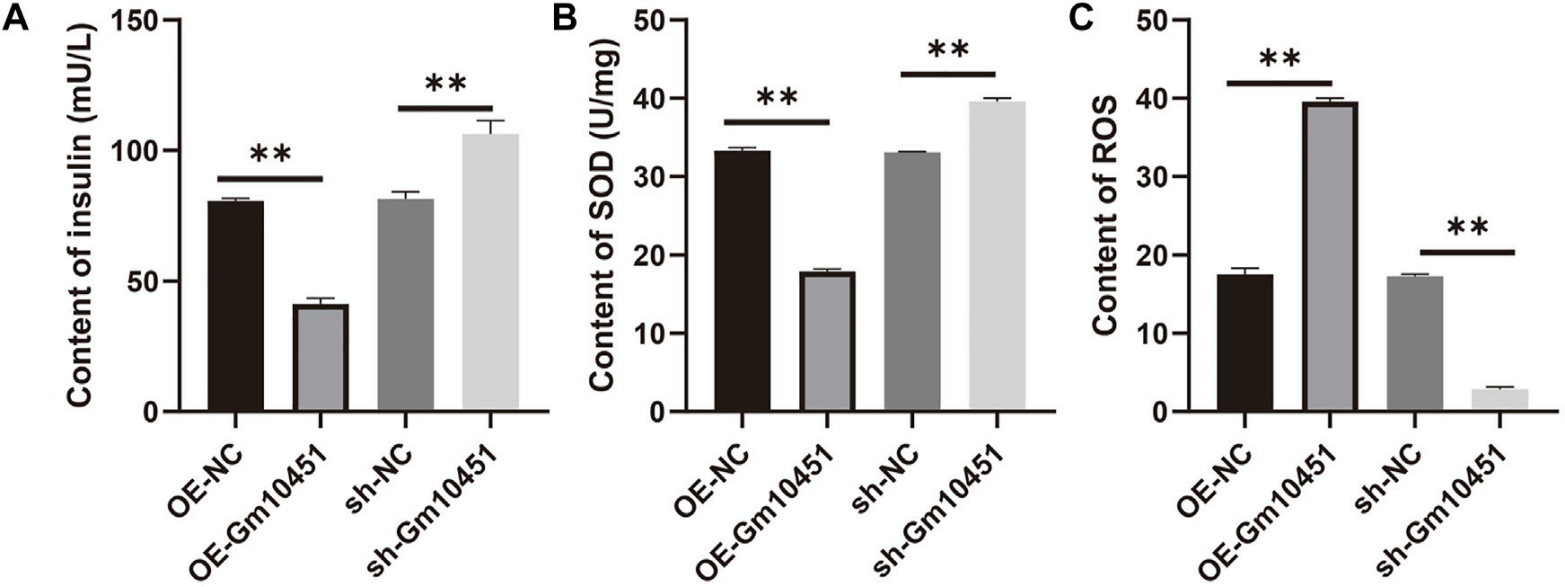
Gm10451 expression affects the insulin secretion and the REDOX reaction of the MIN6 cells cultured in the hyperglycemia environment. **(A)** Determination on the insulin content in MIN6 cells. **(B)** Determination on the SOD content. **(C)** Determination on the ROS content. *N* = 3. **, *p* < 0.01.

In the hyperglycemic environment, transfection of Gm10451 lentivirus into MIN6 cells resulted in decreased insulin secretion, and the difference was statistically significant when compared to the hyperglycemic group (ON-NC group) (*p* < 0.05). Gm10451 siRNA transfection into MIN6 cells promoted insulin secretion in the hyperglycemic environment, and the difference was statistically significant when compared to the hyperglycemic group (sh-NC group) (*p* < 0.05; Figure 5A).

### Different levels of Gm10451 affect the REDOX reaction of the MIN6 cells under hyperglycemia

Compared with the hyperglycemia group, in a hyperglycemia context, the ROS content of MIN6 cells transfected with Gm10451 lentivirus was increased, and their SOD activity was decreased (*p* < 0.05); accordingly, under the same condition, SOD activity of MIN6 cells transfected with Gm10451 siRNA was increased and their ROS content was decreased (Figures 5B,C).

## Discussion

On the base of the current understanding of diabetes pathogenesis, it is certain that insulin resistance and the abnormity in pancreatic *β* cells’ number and function are two major factors in the occurrence and development of diabetes. Further research has shown that diabetes is partly attributed to cell damage, namely, apoptosis of the islet *β* cells. The inflammatory and anti-inflammatory factors released by islet cells include IL-1 and IL-10, and the imbalance of these inflammatory and anti-inflammatory factors secreted may cause islet β-cell damage and lead to its death (Li et al., 2021c; Li et al., 2021a; Tanwar et al., 2021). Functionally, insulin promotes glucose uptake and glycogen synthesis, and the glycogen synthesis is a further factor affecting the absorption and metabolism of blood sugar, which increases insulin sensitivity. In this study, we cultured islet cell MIN6 cells with the hyperglycemic environment and found that the hyperglycemic environment inhibited the proliferation of islet cell MIN6 cells, resulting in enhanced apoptotic activity, increased secretion of inflammatory factors, and decreased secretion of anti-inflammatory factors.

Biological function of lncRNA has not been fully elucidated. It is known that lncRNAs bind the key transcription factors to promoters and regulate target genes’ expression patterns through transcription factors themselves (Ghafouri-Fard et al., 2021; May et al., 2021; Ren et al., 2021). Evidence suggested that Gm10451 is normally expressed in orthopedic diseases, but the function of this lncRNA has not been studied. Through experiments, we found that the expression of Gm10451 in islet *β-*cells was upregulated by hyperglycemia; Gm10451 expression was also upregulated in islet *β-*cells transfected with Gm10451 lentivirus under hyperglycemia. Our experiment also confirmed that continuous hyperglycemia inhibits pancreatic *β-*cell proliferation, increases Caspase 3 activity and inflammatory cytokines secretion, and further inhibits anti-inflammatory cytokines secretion.

In hyperglycemia, transfection of Gm10451 siRNA into islet *β-*cells inhibited the expression of Gm10451 and reversed those changes before. In addition, diabetes is closely linked to oxidative stress, which causes an excess of free radicals such as reactive oxygen species and is responsible for the dynamic imbalance between oxidative and antioxidant systems, which causes inflammation and damage to pancreatic tissue; then reduces the activity of SOD, an important antioxidant enzyme that scavenge oxygen free radicals, exacerbates inflammation and ultimately leads to apoptosis and damage of MIN6 cells (Gong et al., 2021; Kim et al., 2021; Sultan et al., 2021). Our results were sufficient to prove that hyperglycemia promotes ROS production and reduces SOD activity of islet β cells; whereas, in this environment, a downregulation to the Gm10451 expression in pancreatic β-cells improves SOD activity, suppresses ROS content, and alleviates oxidative stress. Therefore, it was reasonably concluded that Gm10451 affects the secretion of oxidative stress and inflammatory factors, which would eventually cause alterations in islet β-cell proliferation and insulin secretion under hyperglycemia.

This study also has some limitations. The regulatory function of Gm10451 was not verified in animal models or clinical samples. In future studies, we will construct an animal model of diabetes and conduct further validation studies by injecting Gm10451 adenovirus.

## Conclusion

High expression of Gm10451 in the hyperglycemic environment promotes the apoptosis of islet cells, and it also produces oxidative stress as well as inflammatory response in islet cells. However, a purposive inhibition toward Gm10451 expression in the same context enhances islet cell proliferation and improves insulin secretion.

## Data Availability

The datasets presented in this study can be found in online repositories. The names of the repository/repositories and accession number(s) can be found in the article/Supplementary Material.
